# Genome-Wide Analysis and Expression Profiling of the *SUC* and *SWEET* Gene Families of Sucrose Transporters in Oilseed Rape (*Brassica napus* L.)

**DOI:** 10.3389/fpls.2016.01464

**Published:** 2016-09-28

**Authors:** Hongju Jian, Kun Lu, Bo Yang, Tengyue Wang, Li Zhang, Aoxiang Zhang, Jia Wang, Liezhao Liu, Cunmin Qu, Jiana Li

**Affiliations:** Chongqing Engineering Research Center for Rapeseed, College of Agronomy and Biotechnology, Southwest UniversityChongqing, China

**Keywords:** *Brassica napus*, sucrose transporters, SUC, SWEET, expression analysis, stresses response

## Abstract

Sucrose is the principal transported product of photosynthesis from source leaves to sink organs. SUTs/SUCs (sucrose transporters or sucrose carriers) and SWEETs (Sugars Will Eventually be Exported Transporters) play significant central roles in phloem loading and unloading. SUTs/SUCs and SWEETs are key players in sucrose translocation and are associated with crop yields. The SUT/SUC and SWEET genes have been characterized in several plant species, but a comprehensive analysis of these two gene families in oilseed rape has not yet been reported. In our study, 22 and 68 members of the SUT/SUCs and SWEET gene families, respectively, were identified in the oilseed rape (*Brassica napus*) genome through homology searches. An analysis of the chromosomal distribution, phylogenetic relationships, gene structures, motifs and the cis-acting regulatory elements in the promoters of *BnSUC* and *BnSWEET* genes were analyzed. Furthermore, we examined the expression of the 18 *BnSUC* and 16 *BnSWEET* genes in different tissues of “ZS11” and the expression of 9 *BnSUC* and 7 *BnSWEET* genes in “ZS11” under various conditions, including biotic stress (*Sclerotinia sclerotiorum*), abiotic stresses (drought, salt and heat), and hormone treatments (abscisic acid, auxin, cytokinin, brassinolide, gibberellin, and salicylic acid). In conclusion, our study provides the first comprehensive analysis of the oilseed rape SUC and SWEET gene families. Information regarding the phylogenetic relationships, gene structure and expression profiles of the SUC and SWEET genes in the different tissues of oilseed rape helps to identify candidates with potential roles in specific developmental processes. Our study advances our understanding of the important roles of sucrose transport in oilseed rape.

## Introduction

Sucrose is the principal transported product of photosynthesis from source leaves to sink organs (Peng et al., 2014). Sucrose transporters/sucrose carriers (SUTs/SUCs) and SWEETs (Sugars Will Eventually be Exported Transporters) play significant central roles in phloem loading and unloading (Kuhn and Grof, 2010; Chen et al., 2012).

The first SUC was identified in spinach (*Spinacia oleracea* L.) (Riesmeier et al., 1992). Until now, cDNAs for SUCs have been isolated in almost all higher plants (Sauer and Stolz, 1994; Barker et al., 2000; Aoki et al., 2002). Previous studies have revealed that SUCs are located in the plasma membranes of the sieve element and companion cells and are expressed in various tissues from source leaves to sink tissues (Aoki et al., 2004; Sauer et al., 2004; Sivitz et al., 2005; Liesche et al., 2008; Tang et al., 2010). Phylogenetic analysis has indicated that SUCs can be divided into three types based on sequence homology and biochemical activity (Aoki et al., 2003). Briefly, type I is eudicot-specific, and functions in phloem loading (Riesmeier et al., 1994; Gottwald et al., 2000) and normal pollen function (Sivitz et al., 2008). Type II and III SUCs are found in all plant species, and monocots utilize type II SUCs for phloem loading (Slewinski et al., 2009). Type III SUCs, characterized as H+-coupled symporters (Weise et al., 2000), are localized to the vacuolar membrane (Endler et al., 2006; Reinders et al., 2008) and function in sucrose uptake into the cytoplasm (Reinders et al., 2008; Schulz et al., 2011), whereas types I and II are localized to the plasma membrane. In *Arabidopsis*, seven SUC-like genes and two pseudogenes have been characterized (Sauer et al., 2004). Five of these SUC-like genes (*AtSUC1/2/5/8/9*) belong to type I, *AtSUC3* is categorized as type II, and *AtSUC4* is grouped to type III. AtSUC2 is the best-characterized type I SUC, and mutational analysis has shown that SUCs are responsible for restraining plant growth and pollen germination (Gottwald et al., 2000; Sivitz et al., 2008; Srivastava et al., 2009). Antisense transformation experiments have also revealed that SUCs are responsible for the retardation of sucrose translocation, fruit size reduction, and reduced fertility in tomato (Riesmeier et al., 1994; Hackel et al., 2006).

Because SUCs load the sieve element companion cell complex with sucrose from the cell wall space, sucrose exits the symplasm with the help of sucrose transporting SWEETs (Chen et al., 2012). In marked contrast to SUCs, the newly identified class of sugar transporters called SWEETs increased our understanding of cellular sugar export. SWEET proteins consist of only seven predicted transmembrane domains (Chen et al., 2010) and have been characterized in plants, animals and humans. A phylogenetic tree of SWEET proteins from 15 plant taxa focusing on angiosperms was constructed by Eom (Kuhn and Grof, 2010). According to this tree, 17 members of the SWEET family in *Arabidopsis thaliana* fell into four phylogenetic clades. According to their phylogenetic relationships, SWEETs 1–3 were in clade I, SWEETs 4–8 in clade II, SWEETs 9–15 in clade III, and SWEETs 16–17 in clade IV. Clade I and II members prefer hexose transporters, and clade III members are efficient sucrose transporters. Clade IV SWEETs in *Arabidopsis* (AtSWEET16–17) are located on the tonoplast membrane and likely transport fructose (Chen et al., 2012; Klemens et al., 2013). AtSWEET8 and 13 feed pollen, and SWEET9 is essential for nectar secretion (Chen et al., 2010; Lin et al., 2014). SWEET11 and 12 provide sucrose to the SUTs for phloem loading. Mutations in either the AtSWEET11 or 12 genes produced no obvious phenotypes, but double mutants (atsweet11; 12) exhibited moderate defects in sucrose phloem transport and an excessive accumulation of carbohydrates in the leaves. GFP fusions indicate that AtSWEET11, 12 and 15 are expressed in the seed coat and endosperm. The triple knockout mutant exhibited a severe delay in embryo development and a wrinkled seed phenotype at maturity because of lower starch and lipid content and a smaller embryo (Lin et al., 2014). The remaining family members await characterization and could be involved in the gametophyte stage and in sugar transport in the plant.

Oilseed rape (*Brassica napus* L.) is a major global oil crop that is used for direct human consumption, as animal feed, and more recently, as a source of biofuel. High seed yield is one of the most important challenges in *B. napus* breeding, whereas the harvest index (HI) is only approximately 0.2–0.3 (Luo et al., 2015). Studies have implicated that the source and sink organs are not limiting, whereas assimilation translocation is the key limiting factor for seed yield in *Brassica* (Shen et al., 2005). *SUC*s and *SWEET*s may be critical genes for increasing seed yield by translocating sucrose from the source to the sink. However, information regarding *SUC* and *SWEET* in *B. napus* is lacking. In *B. napus*, the homologe of *AtSUC1/5, BnSUC1C*, was cloned, and its mRNA expression profiles were determined (Li et al., 2013). The results indicated that *BnSUC1C* is predominantly expressed in the later developmental stage of the anther. In another study, Song et al. (2015) determined the expression patterns of *BnSUC* family members in leaf, flower, silique, and seed development. Unfortunately, no characterized SWEET member in *B. napus* has been reported.

In our study, we used the BLASTP search program to query for SUC and SWEET family members in the genomes of three species, *B. napus, B. rapa*, and *B. oleracea*, using the AtSUC and AtSWEET protein sequences as the query, respectively. Their expression patterns were also determined using unpublished RNA-Seq data from our laboratory. Further analysis provided new insights into the mechanisms and regulation of assimilate allocation and a new potential for increasing crop yield.

## Materials and methods

### Plant materials and treatments

Plants of oilseed rape (*Brassica napus* Zhongshuang11) were grown in field conditions in Chongqing, China. To analyse transcripts of BnSUC/BnSWEET members in different tissues, roots (R), stems (ST), senescent leaves (SL), extended leaves (EL), buds (B), flowers (F), stalk (ST), silique walls (21 DAF, SW), seeds (21 DAF, SE), and main inflorescences (MI) were collected from “ZS11”. All tissues were quickly frozen in liquid nitrogen and stored at −80°C until use.

For analysis of *BnSUC*/*BnSWEET* members under various abiotic or exogenous hormone stresses, oilseed rape seedlings (at the four-leaf stage) were grown in a greenhouse under long-day conditions (16-h light, 8-h dark) and transferred to 1/2 Hoagland solution 24 h before the induction of different stresses. A final concentration of 200 mM NaCl or 20% polyetheleneglycol-6000 (PEG-6000) was used for salt or drought stress, respectively. For heat stress, seedlings were transferred to a growth chamber at 40°C. For *Sclerotinia sclerotiorum* stress, methods were in accord with Li et al. (2015). For hormone treatment, 50 μM ABA (GenTel, Beijing, China), 100 μM GA3 (GenTel, Beijing, China), 10 μM BR (GenTel, Beijing, China), 75 μM NAA (GenTel, Beijing, China), 75 μM 6-BA (GenTel, Beijing, China), and 2 mM SA (GenTel, Beijing, China) were employed (Yang et al., 2009; Gao et al., 2010). Leaves were collected at 0, 3, 6, 12, 24, 48, and 72 h after salt, drought and heat treatments and 0, 0.5, 1, 3, 6, 12, and 24 h after various exogenous hormone treatments and immediately frozen in liquid nitrogen for storage at −80°C until use.

### Identification of sucrose transporters in *B. napus, B. rapa*, and *B. oleracea*

*Brassica rapa* and *Brassica oleracea* sucrose transporters *SUC* and *SWEET* were identified by performing a BLASTP analysis (Altschul et al., 1997) with the BRAD database (http://brassicadb.org/brad/index.php) and the *B. napus* genome (http://www.genoscope.cns.fr/brassicanapus/) at a cut-off value of < *E*^−20^ using the *A. thaliana* sucrose transporter *SUC* and *SWEET* amino acid sequences as the query sequences, respectively.

### Phylogenetic analyses of the *SUC* and *SWEET* families in *B. napus*

To gain insights into the evolutionary relationships of oilseed rape SUC and SWEET proteins, we performed multiple alignments of the SUC and SWEET proteins of certain species (*A. thaliana, B. rapa, B. oleracea*, and *B. napus*). Multiple sequence alignments of the deduced amino acid sequences of the SUC and SWEET proteins were performed using the default parameters of ClustalW (Eom et al., 2015). Dendrograms were generated by the MEGA 6 program (Tamura et al., 2013) using the neighbor-joining (NJ) method and bootstrap analysis (1000 replications).

### Protein properties and sequence analyses

The molecular weight (MW) and isoelectric points (pI) of the presumed sucrose transporter proteins were predicted by the online ExPASy proteomics server database (http://expasy.org/). The Gene Structure Display Server (GSDS 2.0, http://gsds.cbi.pku.edu.cn/index.php) was used to generate the exon/intron organization. Motifs were identified using the MEME program (http://meme-suite.org/). The maximum number of motifs was 25 and 29 in the BnSUC and BnSWEET proteins, respectively, and the optimum width of the motifs was set from 6 to 50. Furthermore, all identified motifs were annotated according to InterProScan (http://www.ebi.ac.uk/Tools/pfa/iprscan/).

### Promoter cis-element analysis of *BnSUC*s and *BnSWEETs*

The promoter sequences (1.5 kb upstream of the translation start site) of the *BnSUC* and BnSWEET genes were obtained from the *B. napus* genome (http://www.genoscope.cns.fr/brassicanapus/). PlantCARE (http://bioinformatics.psb.ugent.be/webtools/plantcare/html/) was used to analyse the *BnSUC* and *BnSWEET* gene promoters and identify their cis-elements (Rombauts et al., 1999; Lescot et al., 2002).

### RNA isolation and real-time quantitative RT-PCR

Total RNA was extracted from all samples with the RNeasy extraction Kit (Invitrogen, Carlsbad, CA, USA). cDNA was synthesized from 1 μg of total RNA using M-MLV transcriptase (TaKaRa Biotechnology, Dalian, China) according to the manufacturer's instructions after the contaminated genomic DNA was removed by DNase I treatment. Real-time PCR was used to determine the expression levels of *BnSUC*s and *BnSWEET*s in different tissues and in response to various stresses. Quantitative real-time PCR was performed according to methods described in Wei et al. (2016). To obtain precise and reproducible results, each sample was replicated three times. As many studies have suggested only SWEET9 through SWEET15 function as sucrose transporters, we only performed expression analysis of *BnSWEET9*–*BnSWEET15* in various tissues and stresses. Because of their high similarities, 18 *BnSUC* and 16 *BnSWEET* gene primers were designed for quantitative RT-PCR analysis (Table S1).

## Results

### Identification and phylogenetic analysis of oilseed rape *SUC*s and *SWEET*s

To identify all putative SUC and SWEET protein sequences in *B. napus, B. rapa*, and *B. oleracea*, BLASTP analysis was conducted. Through this approach, 22, 9, and 8 SUC genes were identified. The 22 predicted BnSUCs proteins ranged from 478 (BnaSUC1- 8) to 540 (BnaSUC3-1) amino acid (aa) residues in length, with an average length of 512 aa. The relative molecular mass varied from 50.91 kDa (BnSUC1-8) to 57.19 kDa (BnSUC3-2). The pI values ranged from 6.06 (BnSUC3-2) to 9.40 (BnSUC4-1), with 17 members exhibiting pI values >7 (Table S1). Sixty-eight, 26 and 16 SWEET proteins were identified in the *B. napus, B. rapa*, and *B. oleracea* genomes, respectively. The 68 predicted BnSWEET proteins ranged from 56 (BnSWEET3-2) to 303 (BnSWEET4-4) aa in length, with an average length of 245.5 aa. The relative molecular mass varied from 6.5 kDa (BnSWEET3-2) to 33.45 kDa (BnSWEET4-4). The pIs ranged from 4.68 (BnSWEET3-2) to 9.75 (BnSWEET10-3), with 92.6% (63) members exhibiting pI values >7, and the other three having pI values < 7 (Table 1).

**Table 1:** **A complete list of 22 BnSUCs and 68 BnSWEETs identified in our study**.

**Isoforms**	**Transcript name**	**At Orthologs**	**location**	**gDNA size (bp)**	**exon**	**CDS size (nts)**	**Peptide residues**	**Theoretical Mw (kDa)**	**Theoretical pI**
BnSUC1-1	BnaA02g15620D	AT1G71880	9086066–9087993	1928	3	1542	514	54.68	9.11
BnSUC1-2	BnaC02g20830D	AT1G71880	17467249–17469184	1936	3	1542	514	54.66	9.12
BnSUC1-3	BnaCnng07770D	AT1G71880	7074433–7076898	2466	4	1545	515	54.95	9.29
BnSUC1-4	BnaA07g23350D	AT1G71880	17563832–17566282	2451	4	1545	515	54.89	9.29
BnSUC1-5	BnaC06g32880D	AT1G71880	32943807–32946018	2212	3	1527	509	54.30	9.12
BnSUC1-6	BnaA07g29680D	AT1G71880	21194709–21196861	2153	3	1527	509	54.26	9.18
BnSUC1-7	BnaA03g52810D	AT1G71880	27576722–27578748	2027	3	1464	488	52.17	9.17
BnSUC1-8	BnaA03g01930D	AT1G71880	878161–881476	3316	3	1434	478	50.91	8.84
BnSUC1-9	BnaC03g02450D	AT1G71880	1166850–1168621	1772	3	1434	478	51.01	8.94
BnSUC2-1	BnaA09g30430D	AT1G22710	22640785–22643252	2468	1	1527	509	54.02	9.19
BnSUC2-2	BnaA07g10320D	AT1G22710	9796654–9798956	2303	4	1542	514	54.54	9.2
BnSUC2-3	BnaC05g17970D	AT1G22710	11689572–11691991	2420	4	1527	509	54.01	9.13
BnSUC2-4	BnaC07g13570D	AT1G22710	19195725–19199241	3517	4	1527	509	54.02	9.26
BnSUC3-1	BnaC07g21980D	AT2G02860	28500559–28504204	3646	15	1620	540	57.17	6.45
BnSUC3-2	BnaA06g33960D	AT2G02860	22480871–22484422	3552	11	1614	538	57.19	6.06
BnSUC3-3	BnaA02g26600D	AT2G02860	19635749–19639697	3949	15	1611	537	56.99	6.7
BnSUC3-4	BnaC08g09590D	AT2G02860	14428796–14432387	3592	15	1608	536	57.10	6.31
BnSUC3-5	BnaC02g34840D	AT2G02860	37456417–37460343	3927	15	1602	534	56.60	6.45
BnSUC4-1	BnaC05g49240D	AT1G09960	148868–151999	3132	5	1524	508	54.40	9.4
BnSUC4-2	BnaA06g05900D	AT1G09960	3253085–3256258	3174	5	1524	508	54.33	9.32
BnSUC4-3	BnaA09g57200D	AT1G09960	4071366–4074299	2934	5	1506	502	53.68	9.27
BnSUC4-4	BnaC08g42460D	AT1G09960	36658345–36660990	2646	5	1506	502	53.69	9.19
BnSWEET1-1	BnaA06g15180D	AT1G21460	8312098–8313578	1481	6	741	247	27.18	9.3
BnSWEET1-2	BnaA08g21340D	AT1G21460	15835212–15836787	1576	6	756	252	27.82	9.27
BnSWEET1-3	BnaC05g16660D	AT1G21460	10472176–10473671	1496	6	741	247	27.11	9.38
BnSWEET1-4	BnaCnng57120D	AT1G21460	56915564–56917074	1511	6	756	252	27.80	9.2
BnSWEET2-1	BnaA01g29190D	AT3G14770	20200361–20203319	2959	6	711	237	26.59	8.77
BnSWEET2-2	BnaA05g24790D	AT3G14770	18487161–18489107	1947	6	711	237	26.64	8.95
BnSWEET2-3	BnaC01g36600D	AT3G14770	35824411–35828099	3689	6	711	237	26.70	8.78
BnSWEET2-4	BnaC05g38830D	AT3G14770	37440396–37442379	1984	6	711	237	26.71	9.1
BnSWEET3-1	BnaA02g10400D	AT5G53190	5337282–5339309	2028	6	780	260	29.33	8.71
BnSWEET3-2	BnaA03g12410D	AT5G53190	5659063–5659230	168	1	168	56	6.50	4.68
BnSWEET3-3	BnaA10g06560D	AT5G53190	4978453–4980539	2087	8	666	222	25.02	9.65
BnSWEET3-4	BnaC02g14520D	AT5G53190	10037114–10038949	1836	6	777	259	29.10	8.74
BnSWEET4-1	BnaA02g29150D	AT3G28007	21327589–21329350	1762	6	738	246	27.42	8.94
BnSWEET4-2	BnaA06g31710D	AT3G28007	21255253–21257209	1957	6	894	298	32.98	9.22
BnSWEET4-3	BnaC02g37180D	AT3G28007	40118654–40120396	1743	6	744	248	27.52	8.93
BnSWEET4-4	BnaC07g24860D	AT3G28007	31082097–31084031	1935	6	909	303	33.45	9.16
BnSWEET5-1	BnaA02g33550D	AT5G62850	24068477–24071619	3143	6	723	241	26.97	8.15
BnSWEET5-2	BnaA02g33560D	AT5G62850	24078449–24079880	1432	6	723	241	26.83	8.14
BnSWEET5-3	BnaA09g06250D	AT5G62850	3084026–3085592	1567	6	723	241	27.24	9.04
BnSWEET5-4	BnaC02g42310D	AT5G62850	44976111–44977490	1380	6	723	241	26.97	8.15
BnSWEET5-5	BnaC02g42320D	AT5G62850	44977576–44981878	4303	7	870	290	32.57	9.47
BnSWEET5-6	BnaC03g51420D	AT5G62850	36004646–36006772	2127	4	630	210	23.76	8.2
BnSWEET5-7	BnaC09g50910D	AT5G62850	62877–64429	1553	6	723	241	27.30	8.85
BnSWEET7-1	BnaA03g24670D	AT4G10850	11890420–11893522	3103	5	657	219	24.08	8.74
BnSWEET7-2	BnaA09g22240D	AT4G10850	14796338–14798167	1830	5	750	250	27.39	9.65
BnSWEET7-3	BnaC03g29210D	AT4G10850	17391771–17393716	1946	5	657	219	24.12	8.98
BnSWEET7-4	BnaC09g25980D	AT4G10850	25761757–25763619	1863	5	750	250	27.28	9.56
BnSWEEET8-1	BnaA04g10120D	AT5G40260	8952550–8954124	1575	6	717	239	26.81	8.97
BnSWEEET8-2	BnaA05g30320D	AT5G40260	20995547–20996412	866	4	558	186	20.67	6.68
BnSWEEET8-3	BnaC04g32240D	AT5G40260	34031333–34033034	1702	6	717	239	26.67	8.9
BnSWEEET8-4	BnaC05g44710D	AT5G40260	40864818–40865684	867	3	405	135	15.14	8.49
BnSWEET9-1	BnaA03g18350D	AT2G39060	8618562–8619794	1233	6	801	267	29.87	9.06
BnSWEET9-2	BnaC03g21870D	AT2G39060	11876287–11877214	928	4	444	148	16.54	6.28
BnSWEET9-3	BnaC08g24820D	AT2G39060	26738828–26740538	1711	6	798	266	29.69	9.17
BnSWEET10-1	BnaA03g13530D	AT5G50790	6164069–6165795	1727	7	819	273	31.21	9.53
BnSWEET10-2	BnaA07g09540D	AT5G50790	9291632–9293087	1456	4	528	176	20.06	9.38
BnSWEET10-3	BnaCnng63490D	AT5G50790	63416342–63417591	1250	6	642	214	24.69	9.75
BnSWEET11-1	BnaA01g20460D	AT3G48740	12330696–12333803	3108	6	861	287	31.71	9.26
BnSWEET11-2	BnaA06g16330D	AT3G48740	9160383–9162906	2524	6	870	290	32.07	9.25
BnSWEET11-3	BnaC01g25700D	AT3G48740	21925428–21927786	2359	6	861	287	31.71	9.21
BnSWEET11-4	BnaC03g52910D	AT3G48740	38007946–38010833	2888	6	858	286	31.59	9.38
BnSWEET11-5	BnaC08g20440D	AT3G48740	23173096–23175651	2556	6	870	290	31.98	9.32
BnSWEET12-1	BnaA06g26320D	AT5G23660	18121212–18123538	2327	6	867	289	31.74	9.15
BnSWEET12-2	BnaA09g05190D	AT5G23660	2551570–2553626	2057	6	834	278	30.56	9.2
BnSWEET12-3	BnaC07g30650D	AT5G23660	35027055–35029034	1980	6	867	289	31.77	9.07
BnSWEET12-4	BnaC09g04760D	AT5G23660	2758879–2760877	1999	6	834	278	30.63	9.21
BnSWEET12-5	BnaCnng70080D	AT5G23660	70121585–70122811	1227	5	582	194	21.75	9.63
BnSWEET13-1	BnaA10g06010D	AT5G50800	4247936–4249895	1960	6	885	295	32.75	9.05
BnSWEET13-2	BnaC09g27000D	AT5G50800	28410030–28412206	2177	6	888	296	32.83	9.31
BnSWEET14-1	BnaA01g14360D	AT4G25010	7253990–7255844	1855	6	822	274	30.10	9.2
BnSWEET14-2	BnaA03g47060D	AT4G25010	24132373–24134004	1632	6	822	274	30.29	9.27
BnSWEET14-3	BnaA08g14560D	AT4G25010	12294666–12296567	1902	6	816	272	29.88	9.27
BnSWEET14-4	BnaC01g16870D	AT4G25010	11552361–11554224	1864	6	822	274	30.08	9.2
BnSWEET14-5	BnaC07g39240D	AT4G25010	40220870–40222490	1621	6	822	274	30.29	9.26
BnSWEET14-6	BnaC08g11940D	AT4G25010	17237549–17239366	1818	5	867	289	32.23	9.13
BnSWEET15-1	BnaA02g01450D	AT5G13170	652461–654586	2126	6	894	298	33.17	8.27
BnSWEET15-2	BnaA03g04230D	AT5G13170	1954244–1956086	1843	6	879	293	32.85	8.16
BnSWEET15-3	BnaA10g20120D	AT5G13170	14126353–14129056	2704	6	897	299	33.43	8.15
BnSWEET15-4	BnaC02g04530D	AT5G13170	2368626–2370739	2114	6	894	298	33.14	8.56
BnSWEET15-5	BnaC03g71480D	AT5G13170	178352–180234	1883	6	876	292	32.68	8.15
BnSWEET15-6	BnaC09g43920D	AT5G13170	45016477–45018744	2268	6	897	299	33.39	8.39
BnSWEET16-1	BnaA01g27670D	AT3G16690	19317932–19319707	1776	6	696	232	25.75	8.69
BnSWEET16-2	BnaA03g34260D	AT3G16690	16697377–16698971	1595	6	696	232	25.68	9.06
BnSWEET16-3	BnaC01g35200D	AT3G16690	34490741–34491677	937	3	426	142	15.63	9.37
BnSWEET16-4	BnaC03g39730D	AT3G16690	24734107–24736503	2397	6	696	232	25.88	8.9
BnSWEET17-1	BnaA03g42260D	AT4G15920	21208833–21211979	3147	6	723	241	26.45	8.76
BnSWEET17-2	BnaC03g22520D	AT4G15920	12441183–12442189	1007	4	468	156	17.13	9.12
BnSWEET17-3	BnaC07g33320D	AT4G15920	36585547–36589984	4438	6	723	241	26.50	8.43

**Figure 1:**
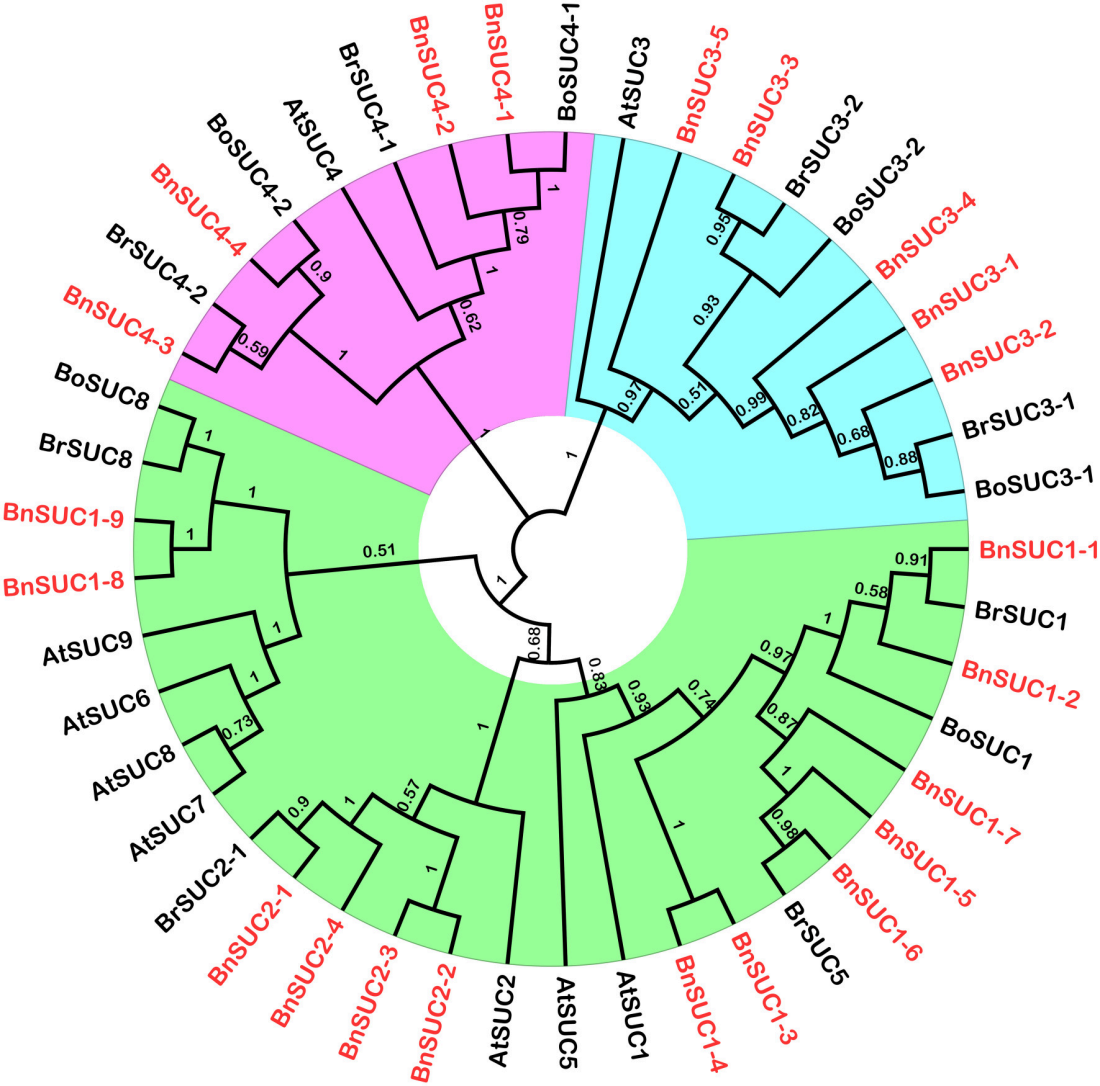
**The phylogenetic analysis of SUC proteins from ***A. thaliana, B. oleracea, B. rape*** and ***B. napus*****. Forty-five SUCs were used to construct the NJ tree with 1000 bootstraps based on the protein sequences. The SUC proteins were grouped into three distinct types.

**Figure 2:**
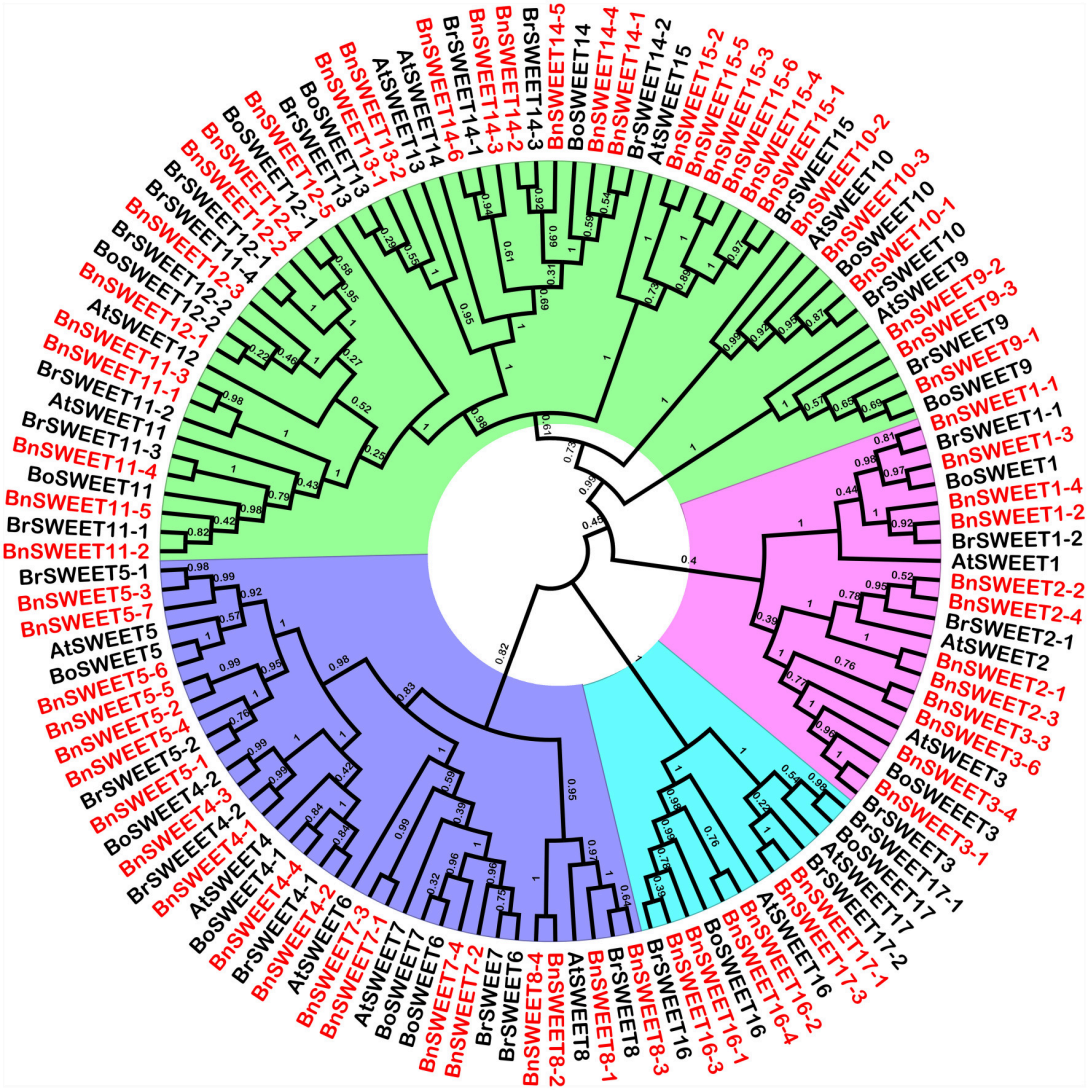
**The phylogenetic analysis of SWEET proteins from ***A. thaliana, B. oleracea, B. rape*** and ***B. napus*****. One hundred and twenty-six. SWEETs proteins were used to construct the NJ tree with 1000 bootstraps based on the protein sequences. The SWEET proteins were clustered into four clades.

To study the evolutionary relationships among oilseed rape SUC and SWEET proteins and known SUCs and SWEETs from *A. thaliana, B. rapa* and *B. oleracea*, an unrooted neighbor-joining phylogenetic tree was created using the amino acid sequences of the SUC and SWEET family proteins from oilseed rape, *A. thaliana, B. rapa*, and *B. oleracea*. Based on our multiple sequence alignment and phylogenetic analysis, SUCs cluster into three types, consistent with previous studies using angiosperms SUCs (Figure 1). Briefly, 26 SUC proteins, including 13 BnSUCs, seven AtSUCs, four BrSUCs, and two BoSUCs, were most likely type I SUCs. AtSUC3, five BnSUC3s, two BrSUC3s, and two BoSUC3s belonged to type II. Type III consisted of four BnSUC4s, two BrSUC4s, two BoSUC4s, and AtSUC4. Although the SWEET gene family is large, 126 members from *A. thaliana, B. napus, B. rapa*, and *B. oleracea* were clustered into four clades (I, II, III, and IV), containing 21, 36, 56, and 13 members, respectively (Figure 2).

### The chromosomal location, gene structure, and conserved motifs of oilseed rape *SUC*s and *SWEET*s

**Figure 3:**
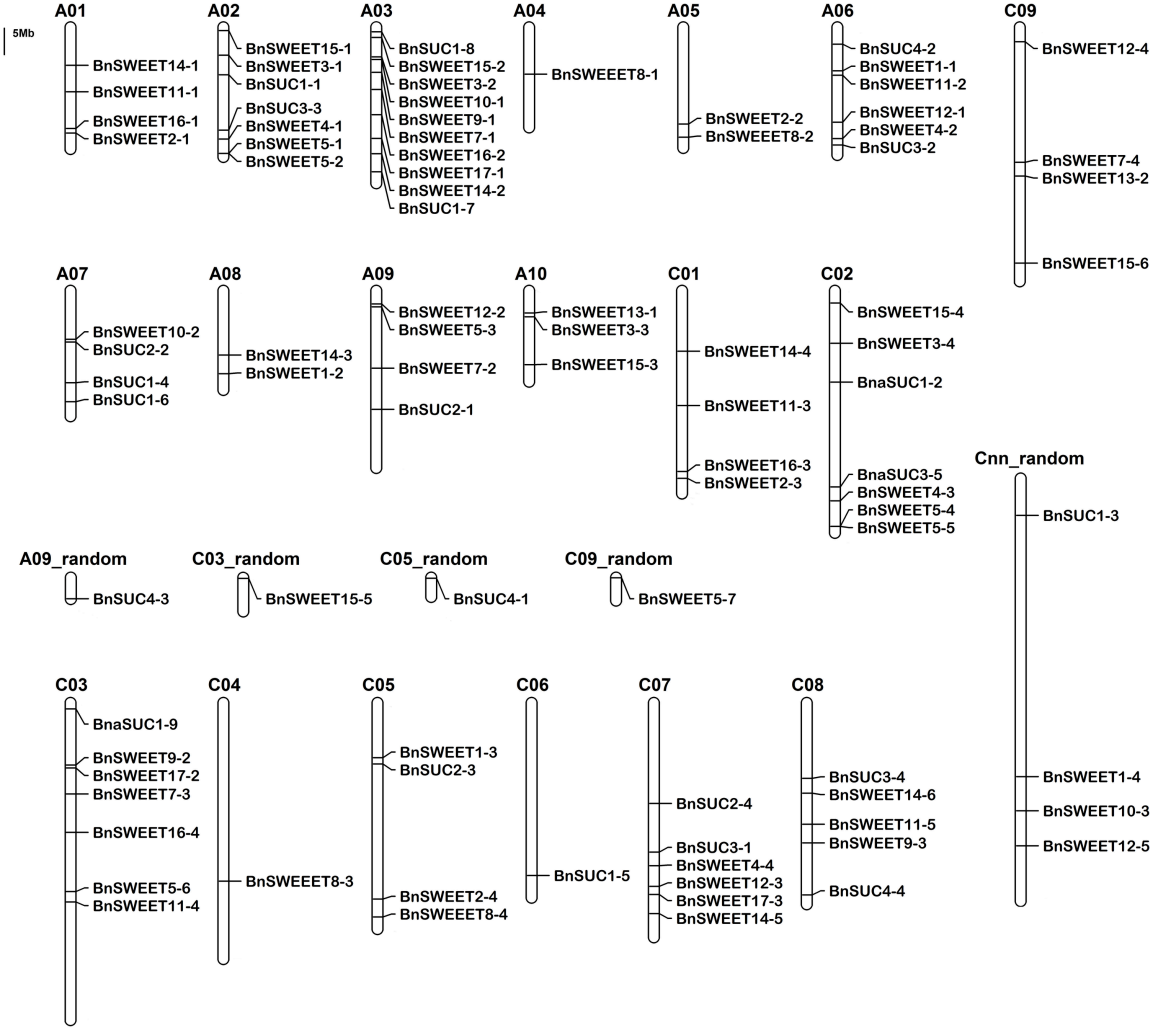
**The distribution of the ***BnSUC*** and ***BnSWEET*** genes in the ***B. napus*** genome**. The chromosomal position of each *BnSUC* and *BnSWEET* gene was mapped according to the *B. napus* genome. The chromosome number is indicated at the top of each chromosome. The scale is in megabases (Mb).

The locations of the *BnSUC* and *BnSWEET* genes are shown in Figure 3. Approximately 50% of the two gene families are located on the A or C genomes, and the precise chromosomal positions of the *BnSUC* and *BnSWEET* genes are listed in Table S1. The 22 *BnSUCs* are located on 14 chromosomes in *B. napus*. Chromosome A07 contains 3 *SUC* genes, whereas chromosomes A02, A03, A06, C02, C07, and C08 each contain two *SUC* genes. Chromosomes A09, C03, C05, C06, A09_random, C05_random and Cnn_random each contain one *SUC* gene (Figure 3). As shown in Figure 3, 68 *BnSWEET* genes are located on A09_random, C09_random, Cnn_random, and on 18 of the 19 chromosomes (the exception is chromosome C06). Chromosome A03 contains the largest number (8) of *BnSWEET* genes, followed by chromosome C03, which contains six members. Chromosome A02 and C02 each contain five members. Five chromosomes each contain four genes, and another five chromosomes (A09, A10, C05, C08, and Cnn_random) each contain three members. Chromosome A05 and A08 each contain two members, and the other five chromosomes (A04, A07, C04, C03_random, and C09_random) each contain only one member.

**Figure 4:**
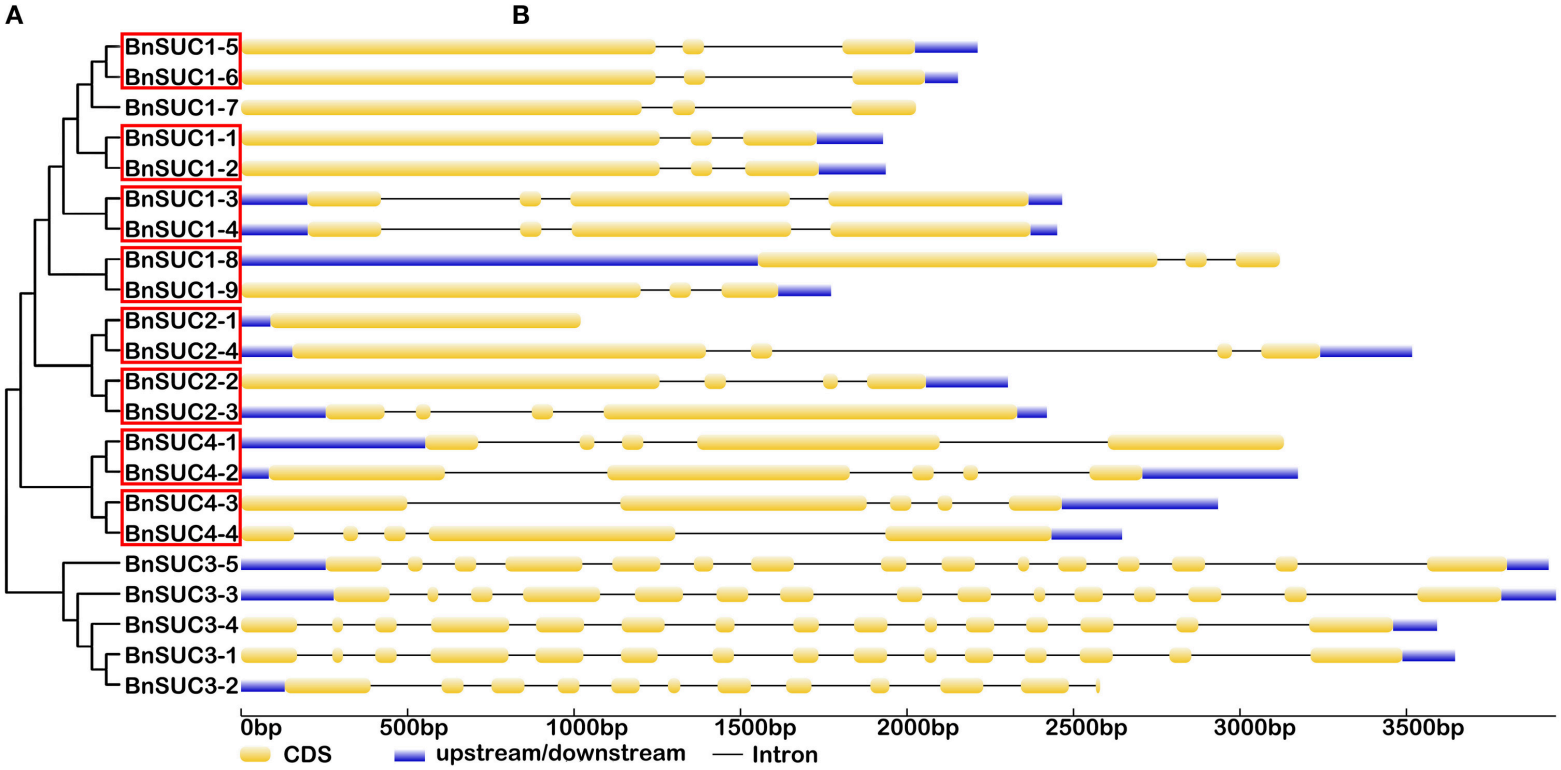
**The exon-intron structure of the ***BnSUC*** genes according to their phylogenetic relationships**. An unrooted phylogenetic tree was constructed with 1000 bootstraps based on the full-length sequences of BnSUC **(A)**. Exon-intron structure analyses of the BnSUC genes were performed using the online tool GSDS **(B)**. The lengths of the exons and introns of each BnSUC gene are proportional. Eight segmental duplicates are highlighted by the red box.

**Figure 5:**
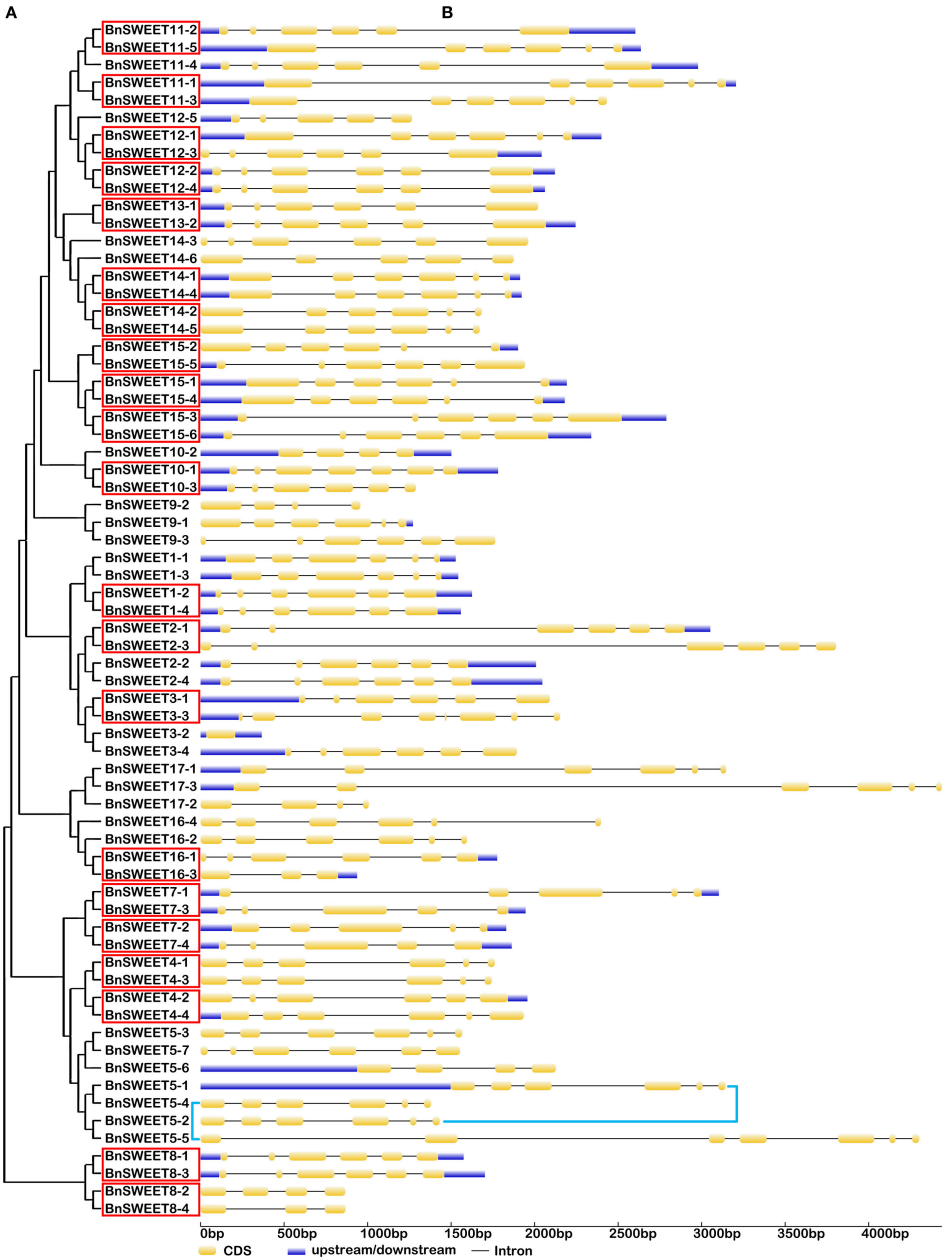
**The exon-intron structure of the BnSWEET genes according to their phylogenetic relationships**. An unrooted phylogenetic tree was constructed with 1000 bootstraps based on the full-length sequences of BnSWEET **(A)**. Exon-intron structure analyses of the BnSWEET genes were performed using the online tool GSDS **(B)**. The lengths of the exons and introns of each BnSWEET gene are proportional. Twenty-one segmental duplicates are highlighted by red boxes. Two tandem duplicates are highlighted by blue lines.

Previous studies have determined that gene divergence and duplication events are the major reasons for evolutionary momentum (Vision et al., 2000; Bowers et al., 2003). In our studies, eight pairs of segmental duplications were identified for the following *BnSUC*s: *BnSUC1-1/BnSUC1-2, BnSUC1-3/BnSUC1-4, BnSUC1-5/BnSUC1-6, BnSUC1-8/BnSUC1-9, BnSUC2-1/BnSUC2-4, BnSUC2-2/BnSUC2-3, BnSUC4-1/BnSUC4-2*, and *BnSUC4-3/BnSUC4-4* (Figure 4). Twenty-three sister gene pairs (including two tandem duplicates: *BnSWEET5-1/BnSWEET5/2* and *BnSWEET5-4/BnSWEET5/5*) were identified for the *BnSWEET* gene family and are shown in Figure 5.

Gene structural diversity and conserved motif divergence play important roles in the evolution of the SWEET/SUC gene family (Hu et al., 2010; Xu et al., 2012). To further examine the structural features of oilseed rape *SUC* and *SWEET* genes, we compared their exon/intron organizations. As shown in Figure 4, the most closely related *BnSUC* genes within the same type shared similar gene structures in terms of either intron numbers or exon lengths. For example, type I genes had one to four exons. Seven of the 13 type I genes had three exons, and the rest had five exons, except *BnSUC2-1*, which had only one exon. By contrast, all type III genes had five exons, and type II members had 15 exons, except *BnSUC3-2*, which had 11 exons. We analyzed the gene structures of *BnSWEET* members using the same approach (Figure 5). Most members (75%) had six exons, and 1, 2, 6, 5, 2, and 1 had 8, 7, 5, 4, 3, and 1 exons, respectively. Overall, 83.3, 57.9, 83.3, and 71.4% of the members in clades I, II, III, and IV contained six exons, respectively.

**Figure 6:**
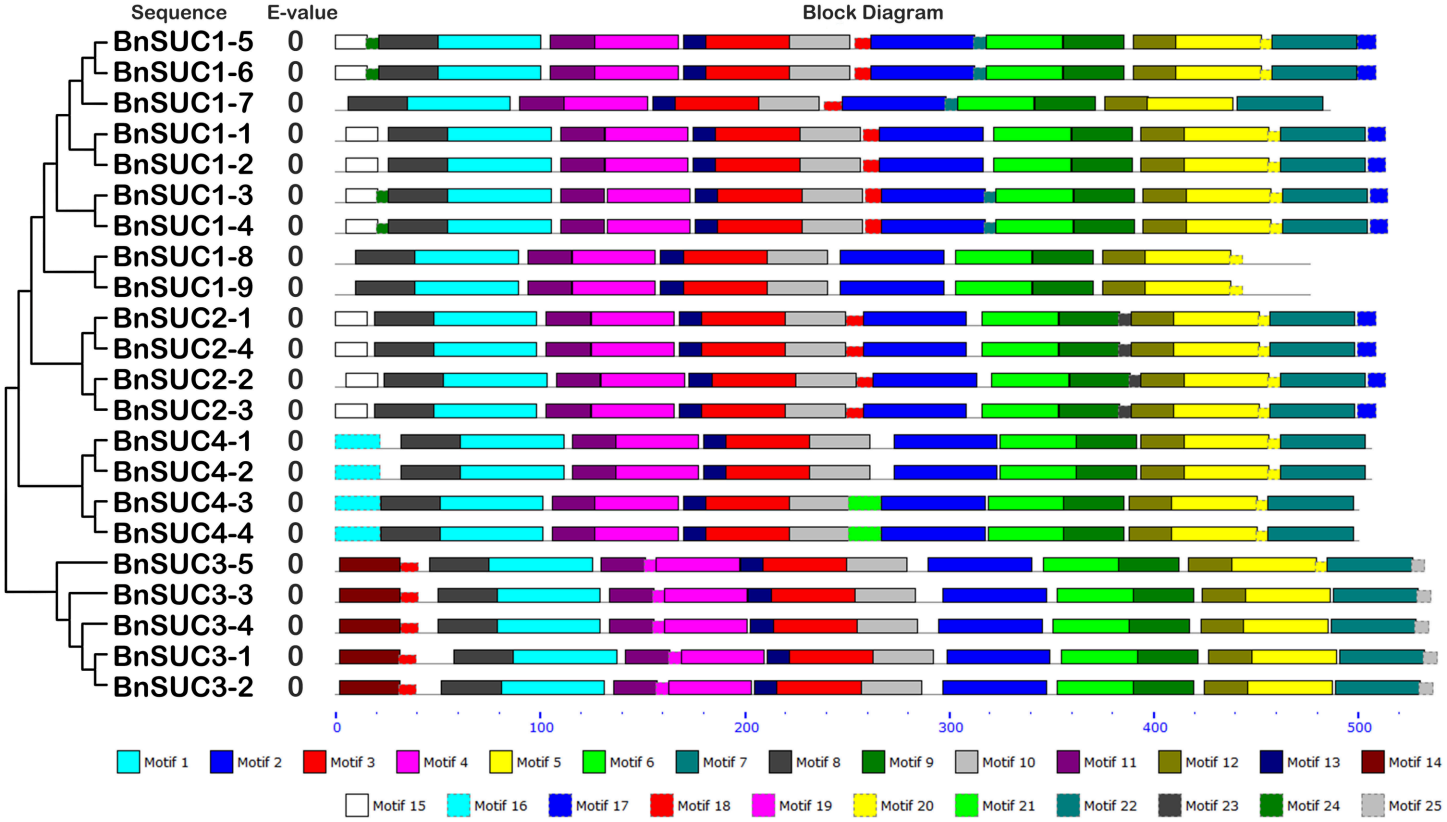
**The conserved motifs of the BnSUC proteins according to their phylogenetic relationships**. The conserved motifs of the BnSUC proteins were identified by MEME. Gray lines represent the non-conserved sequences, and each motif is indicated by a colored box numbered at the bottom. The lengths of motifs in each protein are proportional.

**Figure 7:**
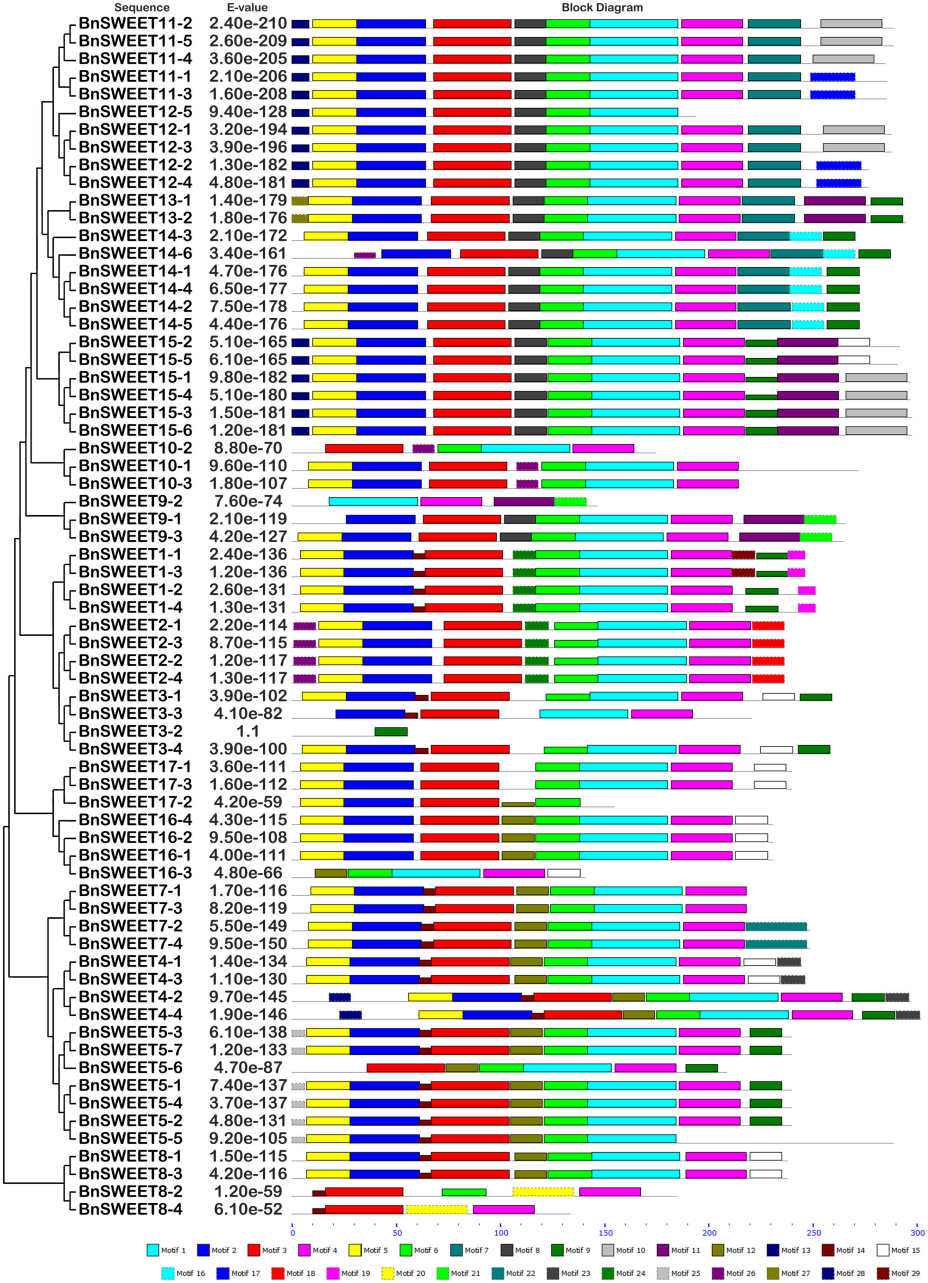
**The conserved motifs of the BnSWEET proteins according to their phylogenetic relationships**. The conserved motifs in the BnSWEET proteins were identified by MEME. Gray lines represent the non-conserved sequences, and each motif is indicated by a colored box numbered at the bottom. The lengths of motifs in each protein are proportional.

Twenty-five and 29 putative protein motifs were predicted using the MEME program for oilseed rape BnSUC (Figure 6) and BnSWEET (Figure 7) proteins, respectively. For BnSUC proteins, motifs 1-13 were observed in all 22 BnSUC proteins. Motifs 15, 17, 22, and 23 were only detected in the type I subgroup. Motifs 14, 19, and 25 were detected only in type II. Motif 16 was only detected in the type III subgroup. According to InterProScan annotation, motifs 1-8 were SUC1-RELATED sucrose transport proteins. Motifs 1-5 were observed in almost all of the BnSWEET proteins and accounted for 92.6, 89.7, 91.2, 92.6, and 85.3% of the motifs, respectively. Motifs 1-3 were annotated as SWEET sugar transporters. Notably, BnSWEET3-2, BnSWEET8-2, BnSWEET8-4, BnSWEET9-2, BnSWEET10-2, BnSWEET16-3, and BnSWEET17-2 were the most diverse, consistent with their gene structures. Generally, proteins with similar motif compositions were clustered in the same class, indicating similar functions among members of the same class.

### The cis-acting regulatory elements in the promoter of *BnSUC*s and *BnSWEET*s

Cis-regulatory elements, which act as binding sites for TFs, are critical for determining the expression patterns of genes (Liu et al., 2013). To further understand the transcriptional regulation and potential functions of *BnSUC*s and *BnSWEETs*, the 1500 bp regions upstream of the transcriptional start codons were analyzed using the PLACE database to identify cis-regulatory elements. Ninety-eight types of cis-regulatory elements were detected in the promoters of *BnSUC* genes, including the TATA-box, CAAT-box, and light responsive elements. In addition, some types of elements are potentially responsive to stresses, including the heat shock element (HSE), the low temperature-responsive element (LTR), the defense and stress-responsive element (TC-rich repeats), the MYB binding site involved in drought-inducibility (MBS), and elements that act in response to hormones, such as the gibberellin-responsive element (GARE-motif), the SA response (TCA-element), the MeJA response (CGTCA motif), and the ABA response (ABRE). All 22 *BnSUC* genes contained 5-19 cis-elements related to stress or hormone responses. Nineteen and 17 *BnSUC* genes contained the GARE motif (gibberellin-responsive element) and the CGTCA motif (MeJA response) in their promoter regions, respectively, suggesting that *BnSUC* genes may play key roles in responses to gibberellin and MeJA. In addition, 18, 16, and 15 *BnSUC* genes contained TC-rich repeats (defense and stress-responsiveness), MBSs (drought stress), and HSEs (heat stress), respectively. Among the 22 BnSUC genes, BnSUC1-2 may be involved in responses to low temperature stress, as implied by the presence of 10 LTR elements (Table S2).

As listed in Table S3, the top seven elements involved in stress and hormone responses were detected in the promoters of *BnSWEET*s. There were 90 instances of the heat shock element (HSE), 103 instances of the defense and stress-responsive element (TC-rich repeats), 80 instances of the MYB binding site involved in drought-inducibility (MBS), 42 instances of the gibberellin-responsive element (GARE-motif), 61 instances of the SA response (TCA-element), 73 instances of the MeJA response (CGTCA motif), and 58 instances of the ABA response element (ABRE).

### Expression profiles of selected *BnSUC* and *BnSWEET* genes in different tissues

**Figure 8:**
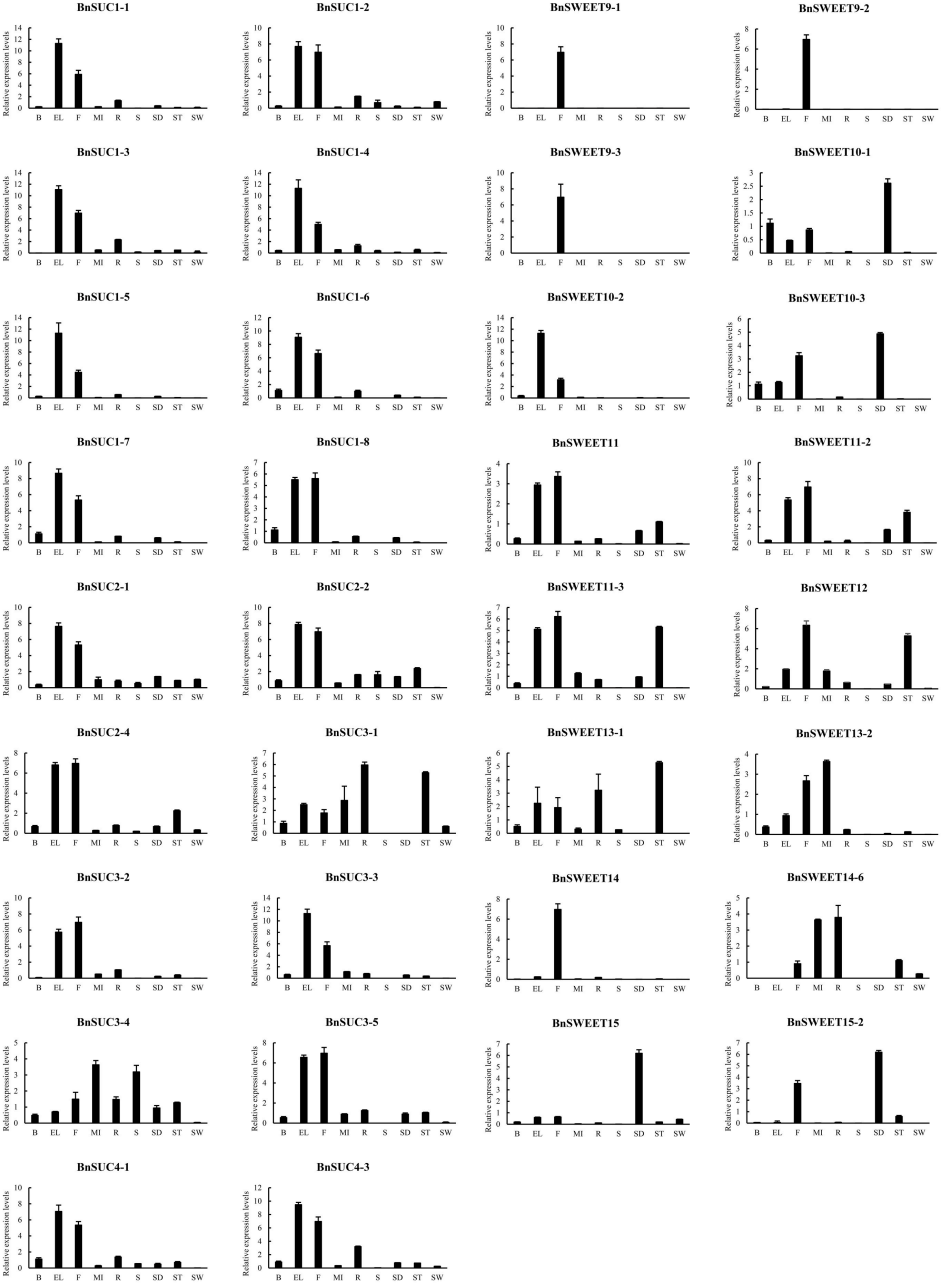
**The tissue-specific expression patterns of selected ***BnSUC*** and ***BnSWEET*** genes in different tissues**. The expression patterns of the selected *BnSUC* and *BnSWEET* genes in the nine indicated organs were analyzed by qRT-PCR. R: roots; S: stems; EL: extended leaves; B: buds; F: flowers; ST: stalk; SW: silique walls; SE: seeds; MI: main inflorescences.

To investigate the functions of *BnSUC and BnSWEET* genes, their gene expression profiles in different tissues were determined by qRT-PCR. Tissues were sampled from roots, stems, senescent leaves, extended leaves, buds, flowers, stalk, silique walls, seeds, and main inflorescences of “ZS11” to explore the expression patterns of selected *BnSUC*s and *BnSWEET*s. As illustrated in Figure 8, the selected *BnSUC* genes including 11 type I, 5 type II, and 2 type III members were mainly expressed in extended leaves and flowers, except 2 type II genes (*BnSUC3-1*, which was mainly expressed in roots and stalks, and *BnSUC3-4*, which was mainly expressed in main inflorescences and stems). Therefore, *BnSUC3* might be involved in different functions with other members.

The expression patterns of selected *BnSWEETs* in different tissues varied. Three *BnSWEET9* genes and all *BnSWEET14* genes were mostly expressed in flowers, indicating that *BnSWEET9* and *BnSWEET14* function in flower development. All *BnSWEET15* members were highly expressed in developing seeds. The rest of the selected *BnSWEET* genes were expressed in a variety of tissues, particularly in extended leaves, developing seeds, stalks and main inflorescences. The three *BnSWEET10* genes had different expression patterns, that is, *BnSWEET10-1*, and *BnSWEET10-3* were mainly expressed in developing seeds, buds, flowers, and extended leaves, whereas *BnSWEET10-2* was highly expressed in extended leaves and flowers but was not expressed in developing seeds and other tissues. Neither BnSWEET11 nor BnSWEET12 expression was detected in stems and silique walls, whereas BnSWEET13-1, and BnSWEET13-2 transcripts were not detected in developing seeds and silique walls. The high expression of *BnSWEET*s in specific tissues may indicate specific roles in the corresponding tissues.

### Response of selected *BnSUC* and *BnSWEET* genes to various abiotic, biotic, and exogenous hormone stresses

**Figure 9:**
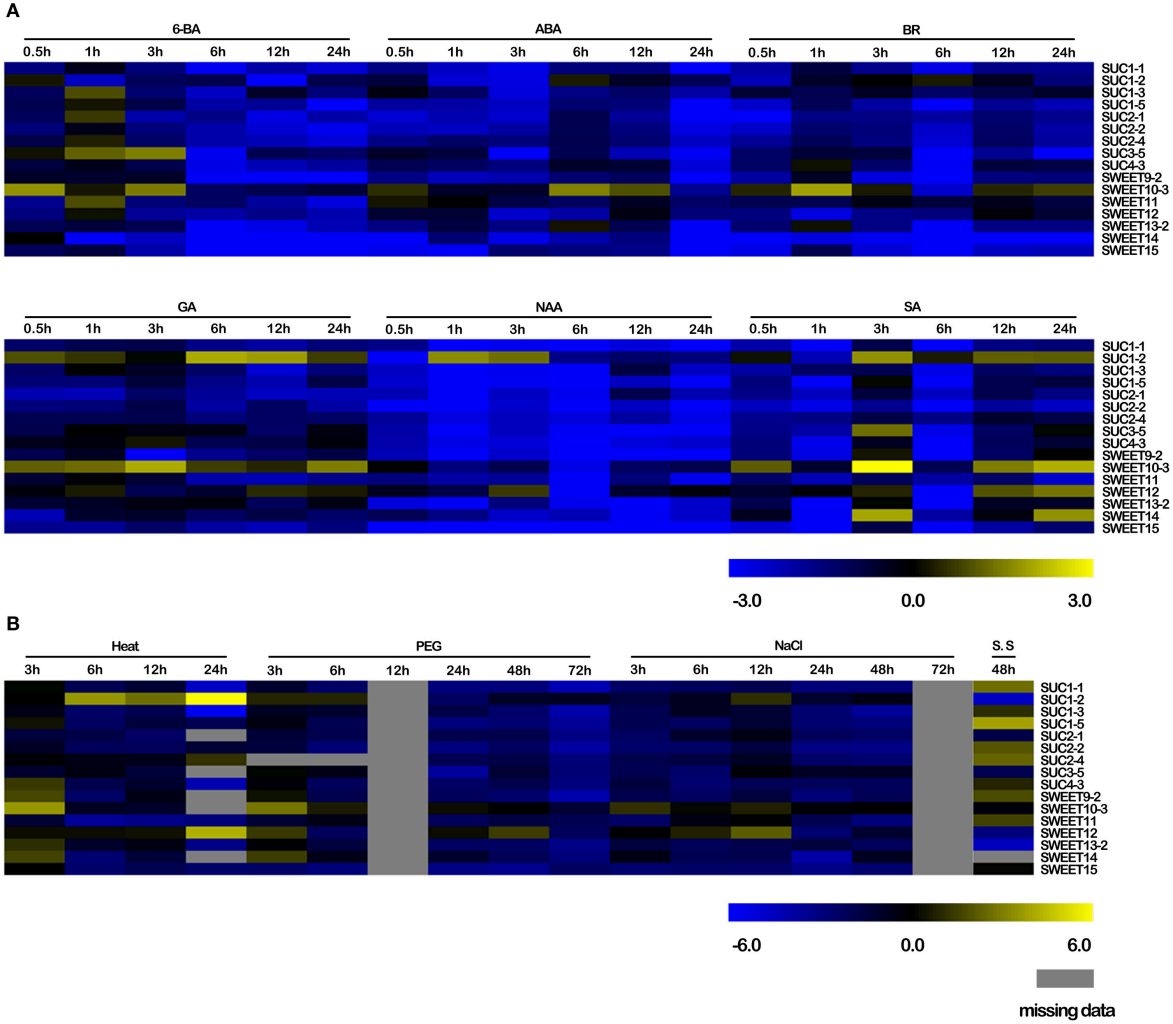
**Responses of selected ***BnSUC*** and ***BnSWEET*** genes to various abiotic, biotic and exogenous hormone stresses**. **(A)** 6-BA, 6-benzylaminopurine, a cytokinin, ABA, abscisic acid, BR, brassinolide, GA, gibberellin, NAA, auxin, SA, salicylic acid, **(B)** Heat, heat stress, NaCl, salt stress, PEG, drought stress, S.S, *Sclerotinia sclerotiorum* stress.

To obtain insight on the roles of *BnSUC* and *BnSWEET* genes responding to various stresses, oilseed rape seedlings of ZS11 were subjected to salt, drought, heat, and S. *sclerotiorum* stresses. As shown in Figure 9, only *BnSUC1-2, BnSWEET10-3* and *BnSWEET12* were upregulated under salt stress, and the remaining genes were downregulated. *BnSUC1-2, BnSWEET10-3, BnSWEET12*, and *BnSWEET14* were upregulated after 3 h under drought stress and then immediately decreased, except *BnSWEET12*. For heat stress, 11 of 16 genes were upregulated after 3 h and decreased immediately, except *BnSUC1-2* and *BnSUC2-4*. For *S. sclerotiorum* infections, *BnSUC1-1, BnSUC1-5, BnSUC2-2, BnSUC2-4, BnSWEET9-2*, and *BnSWEET11* were upregulated after 48 h.

Plant hormones such as SA, auxin, NAA, BR, GA, and ABA play important roles in the regulation of developmental processes (Yang et al., 2012; Curaba et al., 2014). In our study, hormone treatments resulted in a wide variety of changes in the transcript levels of sucrose transporter genes in oilseed rape as determined by qRT-PCR. Briefly, all members were upregulated under GA stress, particularly *BnSUC1-2, BnSUC3-5, BnSUC4-3, BnSWEET10-3, BnSWEET12*, and *BnSWEET13-2*. These genes were also induced by ABA, BR, and 6-BA stresses, whereas only *BnSUC1-2* and *BnSWEET10-3* were induced under SA stress, and only *BnSUC1-2* and *BnSWEET12* were upregulated by NAA stress at some time points (Figure 9). These results indicate that hormones are involved in the regulation of sucrose transporters in oilseed rape.

## Discussion

Sucrose transporters have important functions in plant growth and development, particularly in vascular tissues. Recently, preliminary analyses of the *SUC* and *SWEET* gene families have been conducted in many plant species (Aoki et al., 2004; Hackel et al., 2006; Baker et al., 2012; Chen et al., 2012). The results of these studies shed light on the functions of the *SUC* and *SWEET* genes. However, the *SUC* and *SWEET* gene families have not been studied in *B. napus*, an important oilseed crop. In our study, we analyzed the phylogenetics, intron-exon organization, chromosomal locations, conserved motifs, and expression patterns of the *BnSUC* and *BnSWEET* genes in various tissues and in stress responses.

### The evolution of the oilseed rape *SUC* and *SWEET* genes

Polyploidization is considered an outstanding power of eukaryotic evolution and the main contributor to evolutionary events (Dun et al., 2014). After the *Brassica* genus of plants formed from *A. thaliana*, it triplicated its genome, with an expansion in gene numbers (Lysak et al., 2005, 2007). Generally, three syntenic copies of each gene in *A. thaliana* should be detected in a diploid *Brassica* species, such as *B. rapa* (Wang et al., 2011; Cheng et al., 2013; Dun et al., 2014), and *B. oleracea* (Cheng et al., 2014; Liu et al., 2014). Thus, the allotetraploid *B. napus*, which was generated from the spontaneous hybridization of *B. rapa* and *B. oleracea* approximately 7500–12,500 years ago (Chalhoub et al., 2014), should contain six copies of each *Arabidopsis* gene. However, the *B. rapa* (Mun et al., 2009; Wang et al., 2011), and *B. napus* (Chalhoub et al., 2014; Cheng et al., 2014) genomes only contain 1.5–2 and 2–6 copies of each gene in *Arabidopsis*, respectively, because of genome shrinkage and gene loss. In our study, a total of 22 *SUC* and 68 *SWEET* genes were identified in the genome of the oilseed rape.

Our molecular characterizations revealed great variations. The molecular masses and pIs varied markedly among subfamilies, whereas the SUC and SWEET proteins clustered in the same subfamily and shared closer molecular masses and pIs. Consistent with previous studies (Reinders et al., 2012; Chong et al., 2014), the members of the *SUC* and *SWEET* gene families in the genomes of *A. thaliana, B. napus, B. rapa*, and *B. oleracea* were classified into three and four groups, respectively. Variation in exon–intron structure plays a significant role in the evolution of gene families (Rogozin et al., 2005; Xu et al., 2012). Our studies of the *SUC* and *SWEET* gene families provide an explanation for this diversification in gene structure. Type II BnSUC3s contain more exons than type I and type III BnSUCs, indicating their diverse functions. These data, along with detected motif data, suggest similar origins, and evolutionary patterns for the *SUC* and *SWEET* genes of different species.

### Expression patterns of *SUC* and *SWEET* genes in various tissues and stress responses

Comprehensive gene expression analyses of sucrose transporter family genes have revealed that *SUC*s have distinct expression patterns in various tissues of Arabidopsis, maize, wheat and grape (Meyer et al., 2004; Slewinski et al., 2009; Afoufa-Bastien et al., 2010; Mukherjee et al., 2015). Consistent with these previous results, *BnSUC*s also exhibited differential expression patterns in various tissues. Notably, *BnSUC2* in the type I subclass was highly expressed in different tissues, particularly flowers and extended leaves, suggesting specific roles required in these tissue types. According to previous studies, AtSUC3, which was clustered into type II with the *BnSUC3*s, functions as a sugar signal-transducer. However, SUC1/2 were only observed in eudicot species, and these genes are necessary for phloem loading (Srivastava et al., 2008; Gould et al., 2012) and normal pollen function (Sivitz et al., 2008). Type III SUCs act as sucrose carriers and are localized to the vacuolar membrane (Endler et al., 2006; Schneider et al., 2012).

All clade III members of *BnSWEET*s were highly expressed in the extended leaves, flowers and developing seeds. Similar patterns have been observed in other plant species (Xuan et al., 2013; Lin et al., 2014). Some *BnSWEET*s exhibited high expression levels in specific tissues, implying specific roles required in these tissue types. For example, *BnSWEET9-1/9-2/9-3*, and *BnSWEET14* were highly specifically expressed in flowers, and *BnSWEET15* was abundantly expressed in developing seeds.

Several studies have examined regulators of sucrose transporters. Meyer et al. (2004) observed that *AtSUC3* expression is strongly induced upon wounding of *Arabidopsis* tissues. Chincinska et al. (2008) subsequently determined that *StSUT4* expression in wild-type plants was induced by GA and ET. Mukherjee et al. (2015) demonstrated that ABA negatively regulates sucrose import into the endosperm by repressing TaSUT1 in wheat using physiological, molecular and biochemical approaches. In our study, expression changes in 16 selected genes were detected under heat, drought, salt, *S. sclerotiorum*, and six hormone stresses. Notably, sucrose transporter genes in oilseed rape were positively regulated by cytokinin (6-BA) and GA, consistent with previous reports (Chincinska et al., 2008). By contrast, these selected genes were mostly downregulated under ABA, NAA, SA, NaCl, and PEG stresses. These results indicate that sucrose transporter genes (*BnSUC*s and *BnSWEET*s) are regulated by plant hormones and abiotic and biotic stresses. These results may facilitate improvements in crop yield.

## Author contributions

JL conceived and designed the experiments. HJ, KL, and BY performed the experiments. HJ, KL, BY, TW, LZ, AZ, JW, LL, and CQ analyzed the data. HJ and KL wrote the paper.

### Conflict of interest statement

The authors declare that the research was conducted in the absence of any commercial or financial relationships that could be construed as a potential conflict of interest.

## References

[B1] Afoufa-Bastien D. Medici A. Jeauffre J. Coutos-Thevenot P. Lemoine R. Atanassova R.. (2010). The *Vitis vinifera* sugar transporter gene family: phylogenetic overview and macroarray expression profiling. BMC Plant Biol. 10:245. 10.1186/1471-2229-10-245 21073695 PMC3095327

[B2] Altschul S. F. Madden T. L. Schaffer A. A. Zhang J. Zhang Z. Miller W.. (1997). Gapped BLAST and PSI-BLAST: a new generation of protein database search programs. Nucleic Acids Res. 25, 3389–3402. 9254694 10.1093/nar/25.17.3389 PMC146917

[B3] Aoki N. Hirose T. Scofield G. N. Whitfeld P. R. Furbank R. T. (2003). The sucrose transporter gene family in rice. Plant Cell Physiol. 44, 223–232. 10.1093/pcp/pcg030 12668768

[B4] Aoki N. Scofield G. N. Wang X. D. Patrick J. W. Offler C. E. Furbank R. T. (2004). Expression and localisation analysis of the wheat sucrose transporter TaSUT1 in vegetative tissues. Planta 219, 176–184. 10.1007/s00425-004-1232-7 15014993

[B5] Aoki N. Whitfield P. Hoeren F. Scofield G. Newell K. Patrick J.. (2002). Three sucrose transporter genes are expressed in the developing grain of hexaploid wheat. Plant Mol. Biol. 50, 453–462. 10.1023/A:1019846832163 12369621

[B6] Baker R. F. Leach K. A. Braun D. M. (2012). SWEET as sugar: new sucrose effluxers in plants. Mol. Plant 5, 766–768. 10.1093/mp/sss054 22815540

[B7] Barker L. Kuhn C. Weise A. Schulz A. Gebhardt C. Hirner B.. (2000). SUT2, a putative sucrose sensor in sieve elements. Plant Cell 12, 1153–1164. 10.1105/tpc.12.7.1153 10899981 PMC149056

[B8] Bowers J. E. Chapman B. A. Rong J. Paterson A. H. (2003). Unravelling angiosperm genome evolution by phylogenetic analysis of chromosomal duplication events. Nature 422, 433–438. 10.1038/nature01521 12660784

[B9] Chalhoub B. Denoeud F. Liu S. Parkin I. A. Tang H. Wang X.. (2014). Plant genetics. Early allopolyploid evolution in the post-Neolithic *Brassica napus* oilseed genome. Science 345, 950–953. 10.1126/science.1253435 25146293

[B10] Chen L. Q. Hou B. H. Lalonde S. Takanaga H. Hartung M. L. Qu X. Q.. (2010). Sugar transporters for intercellular exchange and nutrition of pathogens. Nature 468, 527–532. 10.1038/nature09606 21107422 PMC3000469

[B11] Chen L. Q. Qu X. Q. Hou B. H. Sosso D. Osorio S. Fernie A. R.. (2012). Sucrose efflux mediated by SWEET proteins as a key step for phloem transport. Science 335, 207–211. 10.1126/science.1213351 22157085

[B12] Cheng F. Mandakova T. Wu J. Xie Q. Lysak M. A. Wang X. (2013). Deciphering the diploid ancestral genome of the Mesohexaploid *Brassica rapa*. Plant Cell 25, 1541–1554. 10.1105/tpc.113.110486 23653472 PMC3694691

[B13] Cheng F. Wu J. Wang X. (2014). Genome triplication drove the diversification of *Brassica plants*. Hortic. Res. 1:14024. 10.1038/hortres.2014.24 26504539 PMC4596316

[B14] Chincinska I. A. Liesche J. Krugel U. Michalska J. Geigenberger P. Grimm B.. (2008). Sucrose transporter StSUT4 from potato affects flowering, tuberization, and shade avoidance response. Plant Physiol. 146, 515–528. 10.1104/pp.107.112334 18083796 PMC2245842

[B15] Chong J. Piron M. C. Meyer S. Merdinoglu D. Bertsch C. Mestre P. (2014). The SWEET family of sugar transporters in grapevine: VvSWEET4 is involved in the interaction with *Botrytis cinerea*. J. Exp. Bot. 65, 6589–6601. 10.1093/jxb/eru375 25246444

[B16] Curaba J. Singh M. B. Bhalla P. L. (2014). miRNAs in the crosstalk between phytohormone signalling pathways. J. Exp. Bot. 65, 1425–1438. 10.1093/jxb/eru002 24523503

[B17] Dun X. Shen W. Hu K. Zhou Z. Xia S. Wen J.. (2014). Neofunctionalization of duplicated Tic40 genes caused a gain-of-function variation related to male fertility in *Brassica oleracea* lineages. Plant Physiol. 166, 1403–1419. 10.1104/pp.114.246470 25185122 PMC4226349

[B18] Endler A. Meyer S. Schelbert S. Schneider T. Weschke W. Peters S. W.. (2006). Identification of a vacuolar sucrose transporter in barley and *Arabidopsis* mesophyll cells by a tonoplast proteomic approach. Plant Physiol. 141, 196–207. 10.1104/pp.106.079533 16581873 PMC1459324

[B19] Eom J. S. Chen L. Q. Sosso D. Julius B. T. Lin I. W. Qu X. Q.. (2015). SWEETs, transporters for intracellular and intercellular sugar translocation. Curr. Opin. Plant Biol. 25, 53–62. 10.1016/j.pbi.2015.04.005 25988582

[B20] Gao Y. Li T. Liu Y. Ren C. Zhao Y. Wang M. (2010). Isolation and characterization of gene encoding G protein α subunit protein responsive to plant hormones and abiotic stresses in *Brassica napus*. Mol. Biol. Rep. 37, 3957–3965. 10.1007/s11033-010-0054-x 20238175

[B21] Gottwald J. R. Krysan P. J. Young J. C. Evert R. F. Sussman M. R. (2000). Genetic evidence for the in planta role of phloem-specific plasma membrane sucrose transporters. Proc. Natl. Acad. Sci. U.S.A. 97, 13979–13984. 10.1073/pnas.250473797 11087840 PMC17686

[B22] Gould N. Thorpe M. R. Pritchard J. Christeller J. T. Williams L. E. Roeb G.. (2012). AtSUC2 has a role for sucrose retrieval along the phloem pathway: evidence from carbon-11 tracer studies. Plant Sci. 188, 97–101. 10.1016/j.plantsci.2011.12.018 22525249

[B23] Hackel A. Schauer N. Carrari F. Fernie A. R. Grimm B. Kuhn C. (2006). Sucrose transporter LeSUT1 and LeSUT2 inhibition affects tomato fruit development in different ways. Plant J. 45, 180–192. 10.1111/j.1365-313X.2005.02572.x 16367963

[B24] Hu R. Qi G. Kong Y. Kong D. Gao Q. Zhou G. (2010). Comprehensive analysis of NAC domain transcription factor gene family in *Populus trichocarpa*. BMC Plant Biol. 10:145. 10.1186/1471-2229-10-145 20630103 PMC3017804

[B25] Klemens P. A. Patzke K. Deitmer J. Spinner L. Le Hir R. Bellini C.. (2013). Overexpression of the vacuolar sugar carrier AtSWEET16 modifies germination, growth, and stress tolerance in *Arabidopsis*. Plant Physiol. 163, 1338–1352. 10.1104/pp.113.224972 24028846 PMC3813654

[B26] Kuhn C. Grof C. P. (2010). Sucrose transporters of higher plants. Curr. Opin. Plant Biol. 13, 288–298. 10.1016/j.pbi.2010.02.001 20303321

[B27] Lescot M. Dehais P. Thijs G. Marchal K. Moreau Y. Van de Peer Y.. (2002). PlantCARE, a database of plant cis-acting regulatory elements and a portal to tools for *in silico* analysis of promoter sequences. Nucleic Acids Res. 30, 325–327. 10.1093/nar/30.1.325 11752327 PMC99092

[B28] Li F. Yan L. Lai J. Ma C. Gautam M. Fu T. (2013). Molecular cloning and mRNA expression profile of sucrose transporter gene BnSUT1C from *Brassica napus* L. Indian J. Exp. Biol. 51, 1130–1136. 24579380

[B29] Li J. Zhao Z. Hayward A. Cheng H. Fu D. (2015). Integration analysis of quantitative trait loci for resistance to *Sclerotinia sclerotiorum* in *Brassica napus*. Euphytica 205, 483–489. 10.1007/s10681-015-1417-0

[B30] Liesche J. Schulz A. Krugel U. Grimm B. Kuhn C. (2008). Dimerization and endocytosis of the sucrose transporter StSUT1 in mature sieve elements. Plant Signal. Behav. 3, 1136–1137. 10.4161/psb.3.12.7096 19704459 PMC2634480

[B31] Lin I. W. Sosso D. Chen L. Q. Gase K. Kim S. G. Kessler D.. (2014). Nectar secretion requires sucrose phosphate synthases and the sugar transporter SWEET9. Nature 508, 546–549. 10.1038/nature13082 24670640

[B32] Liu S. Liu Y. Yang X. Tong C. Edwards D. Parkin I. A.. (2014). The *Brassica oleracea* genome reveals the asymmetrical evolution of polyploid genomes. Nat. Commun. 5:3930. 10.1038/ncomms4930 24852848 PMC4279128

[B33] Liu Y. Wang L. Xing X. Sun L. Pan J. Kong X.. (2013). ZmLEA3, a multifunctional group 3 LEA protein from maize (Zea mays L.), is involved in biotic and abiotic stresses. Plant Cell Physiol. 54, 944–959. 10.1093/pcp/pct047 23543751

[B34] Luo X. Ma C. Z. Yue Y. Hu K. N. Li Y. Y. Duan Z. Q.. (2015). Unravelling the complex trait of harvest index in rapeseed (*Brassica napus* L.) with association mapping. BMC Genomics 16:379. 10.1186/s12864-015-1607-0 25962630 PMC4427920

[B35] Lysak M. A. Cheung K. Kitschke M. Bures P. (2007). Ancestral chromosomal blocks are triplicated in *Brassiceae* species with varying chromosome number and genome size. Plant Physiol. 145, 402–410. 10.1104/pp.107.104380 17720758 PMC2048728

[B36] Lysak M. A. Koch M. A. Pecinka A. Schubert I. (2005). Chromosome triplication found across the tribe *Brassiceae*. Genome Res. 15, 516–525. 10.1101/gr.3531105 15781573 PMC1074366

[B37] Meyer S. Lauterbach C. Niedermeier M. Barth I. Sjolund R. D. Sauer N. (2004). Wounding enhances expression of AtSUC3, a sucrose transporter from *Arabidopsis* sieve elements and sink tissues. Plant Physiol. 134, 684–693. 10.1104/pp.103.033399 14739351 PMC344544

[B38] Mukherjee S. Liu A. Deol K. K. Kulichikhin K. Stasolla C. Brule-Babel A.. (2015). Transcriptional coordination and abscisic acid mediated regulation of sucrose transport and sucrose-to-starch metabolism related genes during grain filling in wheat (*Triticum aestivum* L.). Plant Sci. 240, 143–160. 10.1016/j.plantsci.2015.09.010 26475195

[B39] Mun J. H. Kwon S. J. Yang T. J. Seol Y. J. Jin M. Kim J. A.. (2009). Genome-wide comparative analysis of the *Brassica rapa* gene space reveals genome shrinkage and differential loss of duplicated genes after whole genome triplication. Genome Biol. 10:R111. 10.1186/gb-2009-10-10-r111 19821981 PMC2784326

[B40] Peng D. Gu X. Xue L. J. Leebens-Mack J. H. Tsai C. J. (2014). Bayesian phylogeny of sucrose transporters: ancient origins, differential expansion and convergent evolution in monocots and dicots. Front. Plant Sci. 5:615. 10.3389/fpls.2014.00615 25429293 PMC4228843

[B41] Reinders A. Sivitz A. B. Starker C. G. Gantt J. S. Ward J. M. (2008). Functional analysis of LjSUT4, a vacuolar sucrose transporter from *Lotus japonicus*. Plant Mol. Biol. 68, 289–299. 10.1007/s11103-008-9370-0 18618272

[B42] Reinders A. Sivitz A. B. Ward J. M. (2012). Evolution of plant sucrose uptake transporters. Front. Plant Sci. 3:22. 10.3389/fpls.2012.00022 22639641 PMC3355574

[B43] Riesmeier J. W. Willmitzer L. Frommer W. B. (1992). Isolation and characterization of a sucrose carrier cDNA from spinach by functional expression in yeast. EMBO J. 11, 4705–4713. 1464305 10.1002/j.1460-2075.1992.tb05575.x PMC556945

[B44] Riesmeier J. W. Willmitzer L. Frommer W. B. (1994). Evidence for an essential role of the sucrose transporter in phloem loading and assimilate partitioning. EMBO J. 13, 1–7. 8306952 10.1002/j.1460-2075.1994.tb06229.x PMC394773

[B45] Rogozin I. B. Sverdlov A. V. Babenko V. N. Koonin E. V. (2005). Analysis of evolution of exon-intron structure of eukaryotic genes. Brief. Bioinformatics. 6, 118–134. 10.1093/bib/6.2.118 15975222

[B46] Rombauts S. Dehais P. Van Montagu M. Rouze P. (1999). PlantCARE, a plant cis-acting regulatory element database. Nucleic Acids Res. 27, 295–296. 9847207 10.1093/nar/27.1.295 PMC148162

[B47] Sauer N. Ludwig A. Knoblauch A. Rothe P. Gahrtz M. Klebl F. (2004). AtSUC8 and AtSUC9 encode functional sucrose transporters, but the closely related AtSUC6 and AtSUC7 genes encode aberrant proteins in different *Arabidopsis* ecotypes. Plant J. 40, 120–130. 10.1111/j.1365-313X.2004.02196.x 15361146

[B48] Sauer N. Stolz J. (1994). SUC1 and SUC2: two sucrose transporters from *Arabidopsis thaliana*; expression and characterization in baker's yeast and identification of the histidine-tagged protein. Plant J. 6, 67–77. 7920705 10.1046/j.1365-313x.1994.6010067.x

[B49] Schneider S. Hulpke S. Schulz A. Yaron I. Holl J. Imlau A.. (2012). Vacuoles release sucrose via tonoplast-localised SUC4-type transporters. Plant Biol. (Stuttg). 14, 325–336. 10.1111/j.1438-8677.2011.00506.x 21972845

[B50] Schulz A. Beyhl D. Marten I. Wormit A. Neuhaus E. Poschet G.. (2011). Proton-driven sucrose symport and antiport are provided by the vacuolar transporters SUC4 and TMT1/2. Plant J. 68, 129–136. 10.1111/j.1365-313X.2011.04672.x 21668536

[B51] Shen J. X. Fu T. D. Yang G. S. Ma C. Z. Tu J. X. (2005). Genetic analysis of rapeseed self-incompatibility lines reveals significant heterosis of different patterns for yield and oil content traits. Plant Breed. 124, 111–116. 10.1111/j.1439-0523.2004.01069.x

[B52] Sivitz A. B. Reinders A. Ward J. M. (2005). Analysis of the transport activity of barley sucrose transporter HvSUT1. Plant Cell Physiol. 46, 1666–1673. 10.1093/pcp/pci182 16091371

[B53] Sivitz A. B. Reinders A. Ward J. M. (2008). *Arabidopsis* sucrose transporter AtSUC1 is important for pollen germination and sucrose-induced anthocyanin accumulation. Plant Physiol. 147, 92–100. 10.1104/pp.108.118992 18359840 PMC2330317

[B54] Slewinski T. L. Meeley R. Braun D. M. (2009). Sucrose transporter1 functions in phloem loading in maize leaves. J. Exp. Bot. 60, 881–892. 10.1093/jxb/ern335 19181865 PMC2652052

[B55] Song J. Jiang L. Jameson P. E. (2015). Expression patterns of *Brassica napus* genes implicate IPT, CKX, sucrose transporter, cell wall invertase, and amino acid permease gene family members in leaf, flower, silique, and seed development. J. Exp. Bot. 66, 5067–5082. 10.1093/jxb/erv133 25873685 PMC4513924

[B56] Srivastava A. C. Dasgupta K. Ajieren E. Costilla G. McGarry R. C. Ayre B. G. (2009). *Arabidopsis* plants harbouring a mutation in AtSUC2, encoding the predominant sucrose/proton symporter necessary for efficient phloem transport, are able to complete their life cycle and produce viable seed. Ann. Bot. 104, 1121–1128. 10.1093/aob/mcp215 19789176 PMC2766205

[B57] Srivastava A. C. Ganesan S. Ismail I. O. Ayre B. G. (2008). Functional characterization of the *Arabidopsis* AtSUC2 Sucrose/H+ symporter by tissue-specific complementation reveals an essential role in phloem loading but not in long-distance transport. Plant Physiol. 148, 200–211. 10.1104/pp.108.124776 18650401 PMC2528097

[B58] Tamura K. Stecher G. Peterson D. Filipski A. Kumar S. (2013). MEGA6: Molecular Evolutionary Genetics Analysis version 6.0. Mol. Biol. Evol. 30, 2725–2729. 10.1093/molbev/mst197 24132122 PMC3840312

[B59] Tang C. Huang D. Yang J. Liu S. Sakr S. Li H.. (2010). The sucrose transporter HbSUT3 plays an active role in sucrose loading to laticifer and rubber productivity in exploited trees of *Hevea brasiliensis* (para rubber tree). Plant Cell Environ. 33, 1708–1720. 10.1111/j.1365-3040.2010.02175.x 20492551

[B60] Vision T. J. Brown D. G. Tanksley S. D. (2000). The origins of genomic duplications in *Arabidopsis*. Science 290, 2114–2117. 10.1126/science.290.5499.2114 11118139

[B61] Wang X. Wang H. Wang J. Sun R. Wu J. Liu S.. (2011). The genome of the mesopolyploid crop species *Brassica rapa*. Nat. Genet. 43, 1035–1039. 10.1038/ng.919 21873998

[B62] Wei L. Jian H. Lu K. Filardo F. Yin N. Liu L.. (2016). Genome-wide association analysis and differential expression analysis of resistance to Sclerotinia stem rot in *Brassica napus*. Plant Biotechnol. J. 14, 1368–1380. 10.1111/pbi.12501 26563848 PMC11389038

[B63] Weise A. Barker L. Kuhn C. Lalonde S. Buschmann H. Frommer W. B.. (2000). A new subfamily of sucrose transporters, SUT4, with low affinity/high capacity localized in enucleate sieve elements of plants. Plant Cell 12, 1345–1355. 10.1105/tpc.12.8.1345 10948254 PMC149107

[B64] Xu G. Guo C. Shan H. Kong H. (2012). Divergence of duplicate genes in exon-intron structure. Proc. Natl. Acad. Sci. U.S.A. 109, 1187–1192. 10.1073/pnas.1109047109 22232673 PMC3268293

[B65] Xuan Y. H. Hu Y. B. Chen L. Q. Sosso D. Ducat D. C. Hou B. H.. (2013). Functional role of oligomerization for bacterial and plant SWEET sugar transporter family. Proc. Natl. Acad. Sci. U.S.A. 110, E3685–E3694. 10.1073/pnas.1311244110 24027245 PMC3785766

[B66] Yang B. Jiang Y. Rahman M. H. Deyholos M. K. Kav N. N. (2009). Identification and expression analysis of wrky transcription factor genes in canola (*Brassica napus* L.) in response to fungal pathogens and hormone treatments. BMC Plant Biol. 9:68. 10.1186/1471-2229-9-68 19493335 PMC2698848

[B67] Yang X. Zhang X. Yuan D. Jin F. Zhang Y. Xu J. (2012). Transcript profiling reveals complex auxin signalling pathway and transcription regulation involved in dedifferentiation and redifferentiation during somatic embryogenesis in cotton. BMC Plant Biol. 12:110. 10.1186/1471-2229-12-110 22817809 PMC3483692

